# A potential herbal component for the future treatment of fatty liver disease: Geniposide from gardenia

**DOI:** 10.3389/fphar.2025.1610676

**Published:** 2025-05-23

**Authors:** Qinyao Zhang, Ziyan Li, Lina Wang

**Affiliations:** ^1^ College of Basic Medicine, Naval Medical University, Shanghai, China; ^2^ Department of Traditional Chinese Medicine, Naval Medical University, Shanghai, China

**Keywords:** nonalcoholic fatty liver disease, geniposide, therapeutic potential, mechanism, fatty liver disease

## Abstract

Nonalcoholic fatty liver disease (NAFLD) is a multisystemic metabolic disease whose global incidence is increasing annually; the currently available treatment options are limited to lifestyle alterations and symptomatic treatments, such as hepatoprotective treatments. However, it is difficult for most patients to adhere to strict lifestyle interventions for long periods, and lifestyle interventions alone have limited effects in some patients with moderate-to-severe NAFLD. Regarding traditional Chinese medicine (TCM) treatments, Zhi-Zi-Da-Huang decoction and Zhi-Zi-Huang-Qin decoction are widely used classic formulas for NAFLD. In this article, we present a review of the progress in research on the use of geniposide, the glycoside analog of gardenia, in the treatment of NAFLD. As a natural ingredient, geniposide has good safety and tolerability profiles and a high potential to exert pharmacological effects. We discuss various mechanisms of action of geniposide in the treatment of NAFLD, including decreased insulin resistance via the regulation of key molecules, such as insulin receptor substrate 1 (IRS-1) and glucose transporter type 1 (GLUT1), and the alleviation of oxidative stress via activation of the nuclear factor erythroid 2-related factor 2/heme oxygenase-1  (Nrf2/HO-1) signaling pathway. Geniposide also enhances mitochondrial function by inhibiting uncoupling protein 2 (UCP2)-mediated proton leakage and has anti-inflammatory and antiapoptotic effects. There are no previous studies reporting the various pharmacological mechanisms of geniposide in the treatment of NAFLD. Our study provides new insights into the effects and mechanisms of action of geniposide, but its toxicity, especially its hepatotoxicity, requires further investigation. Geniposide is a potential drug for the treatment of NAFLD, and further research and development are warranted.

## 1 Introduction

**FIGURE 1:**
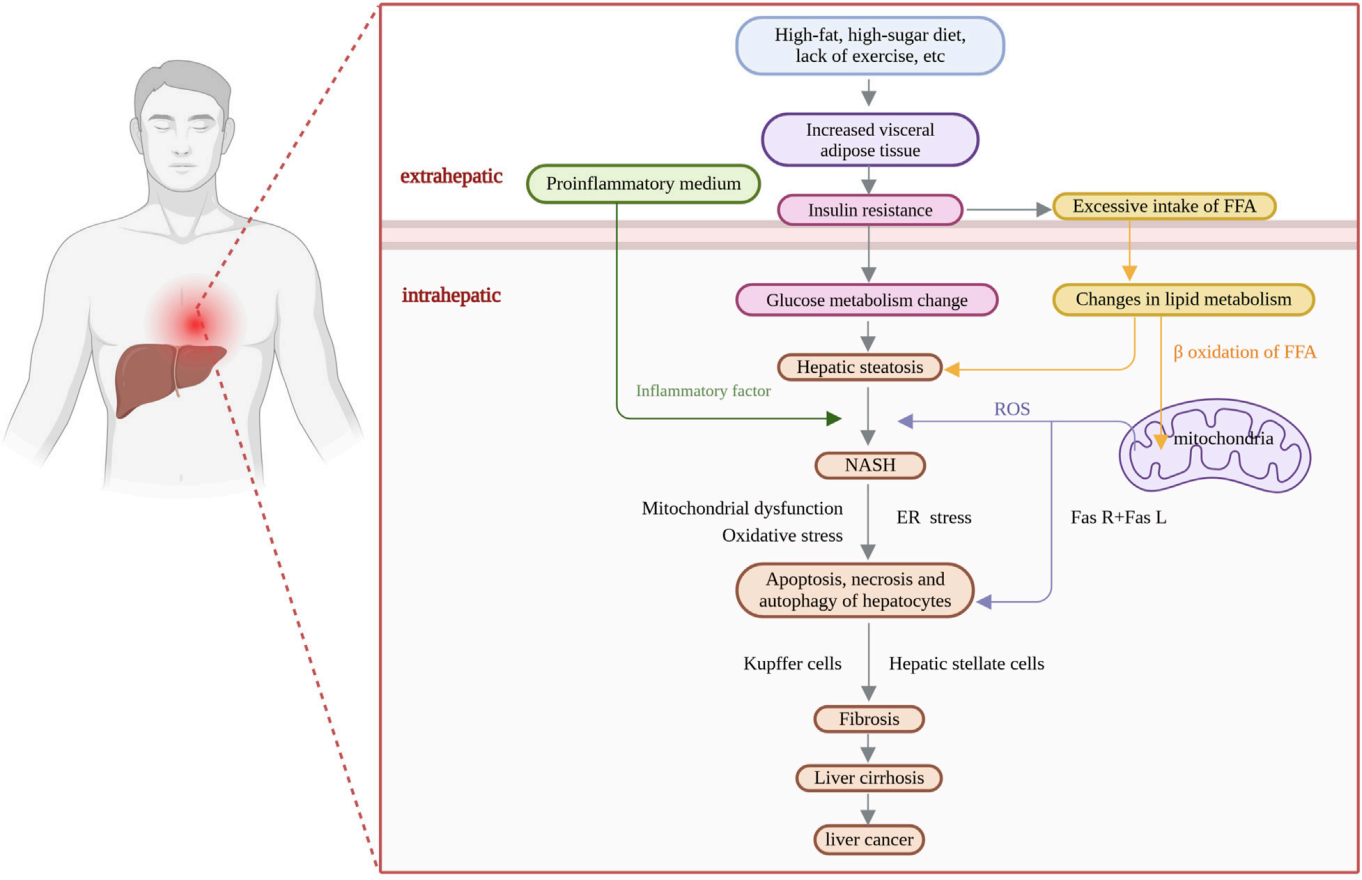
Pathogenesis of nonalcoholic fatty liver disease (NAFLD).

Nonalcoholic fatty liver disease (NAFLD) is a complex multisystemic metabolic disorder that results from the interplay of genetic susceptibility, environmental factors, host metabolic disorders, and changes in the intestinal flora (Singal et al., 2014). The interactions between the host and microbes are reflected in the dysregulation of bile acid production mediated by FXR and FGFR4 (Jiao et al., 2018), as well as the role of alcohol-producing microbiota in vivo (Zhu et al., 2013). As the incidence of NAFLD has continued to increase globally, it has become the leading cause of chronic liver disease; the global prevalence of NAFLD is expected to increase from 25% in 2016 (Younossi et al., 2016) to 55.4% in 2040 (Le et al., 2022). It is expected to become a major factor leading to end-stage liver disease in the next few decades, not only posing a major threat to human health but also imposing a heavy socioeconomic burden (Yip et al., 2023). The pathogenesis of NAFLD is extremely complex (Figure 1); however, the “two-hit” theory is widely accepted by the academic community (Day and James, 1998). Due to in-depth mechanistic studies, “the multiple parallel hits hypothesis”, in which various disease processes (insulin resistance, oxidative stress, mitochondrial stress, inflammatory factor secretion, etc.) occur in parallel, has gradually replaced the traditional “two-hit” theory (Tilg and Moschen, 2010). Although the early stages of NAFLD are characterized by simple steatosis, the disease may progress to steatohepatitis, tissue damage, hepatic fibrosis, cirrhosis, and even hepatocellular carcinoma (Younossi, 2019). Effective control of early-stage NAFLD is achieved mainly through lifestyle modifications, including dietary interventions, increased physical activity and weight loss (Powell et al., 2021). Currently, there is a lack of effective treatment options, and no specific drug has been approved by the FDA for NAFLD (Cassidy and Syed, 2016). Current treatments mainly involve ensuring a balanced diet, weight control, and physical exercise, as well as symptomatic treatments, such as hepatoprotective treatments and reducing blood lipid levels. Bile acid analogs reduce the level of alanine aminotransferase (ALT), but they have many adverse effects and do not lead to the alleviation of histological damage; for example, ursodeoxycholic acid has a hepatoprotective effect in animal models, but it does not ameliorate histological damage to the liver in patients (Chan and Wong, 2019). The combination of pioglitazone and vitamin E has an anti-inflammatory effect but fails to alleviate liver fibrosis (Cheung et al., 2019). Clinical trials of single-target drugs selected on the basis of pathogenic changes—for example, antioxidants, such as peroxisome proliferator-activated receptor (PPAR) agonists, farnesol X receptor (FXR) agonists, and thyroid hormone receptor β (THR-β) agonists—have also yielded unsatisfactory results (Rong et al., 2022).

Traditional Chinese medicine (TCM) formulas have multiple targets and affect multiple pathways. TCM formulas have long been used for the treatment of NAFLD. Zhi-Zi-Da-Huang decoction, Yinchenhao decoction, and Zhi-Zi-Huang-Qin decoction have been widely used in clinical practice as classic formulas for the treatment of NAFLD. Gardenia is the main constituent of the aforementioned formulas and has been shown to clear heat, induce dampness, purge fire and detoxify (Tian et al., 2022). Modern pharmacological studies have confirmed that the main active components of gardenia, such as glycosides and gardenin, reduce steatosis by modulating the inflammatory response and antioxidant activity. Gardenia glycoside, also known as geniposide, is an iridoid glycoside that is not only the active ingredient of gardenia but also found in nearly 40 species of plants, such as *Rehmannia glutinosa*, *Eucommia*, and *Achyranthes*. Numerous pharmacological studies have shown that geniposide has a variety of biological activities, such as hepatoprotective, choleretic, hypoglycemic, hypolipidemic, anti-inflammatory, antioxidant, antidepressant, antitumor, and immunosuppressive activities (Liu et al., 2022). Geniposide suppresses the progression of osteoarthritis by promoting autophagy (Huang et al., 2023), alleviates atherosclerosis by inhibiting lipid phagocytosis (Lin et al., 2024), and alleviates ulcerative colitis (Zhuge et al., 2023). Geniposide is widely used for the treatment of liver diseases. It can inhibit mitochondrial uncoupling protein 2 (UCP2) to combat oxidative stress and inhibit tumor cell proliferation; additionally, it can be used as a PPARγ agonist to prevent the postoperative recurrence of hepatocellular carcinoma (Wu et al., 2023). Geniposide can also inhibit oxidative stress and apoptosis and regulate the metabolism of glycerophospholipids, arginine, and proline to alleviate liver fibrosis (Yang et al., 2021). These findings provide a solid theoretical basis for the development and application of geniposide as a new drug for the treatment of NAFLD.

## 2 Geniposide attenuates insulin resistance (IR)

IR is one of the main causes of NAFLD. In IR, the lipase-inhibiting activity of insulin decreases, and free fatty acids (FFAs) are released in large quantities, which disrupts mitochondrial function and induces toxic effects, leading to hepatocellular steatosis. Excess FFAs are converted to triacylglycerol and accumulate in hepatocytes, aggravating steatosis (Pirgon et al., 2011). IR-induced hyperinsulinemia promotes glycolysis and fatty acid synthesis and inhibits fatty acid β-oxidation, further aggravating lipid deposition in hepatocytes. Therefore, IR is considered the strongest predictor of NAFLD risk (Buzzetti et al., 2016). Studies have shown that the severity of NAFLD is positively correlated with the degree of IR; that is, the more severe NAFLD is, the more pronounced the degree of IR is (Khan et al., 2019).

Oxidative stress is an important factor in IR. Geniposide attenuates IR by suppressing free radical generation and alleviating oxidative stress through antioxidant effects. Lili Guan et al. (Guan et al., 2013) found that geniposide facilitates glucose consumption and glycogen synthesis by stimulating insulin secretion from pancreatic islet cells; restores insulin sensitivity; inhibits cellular reactive oxygen species (ROS) production; slows decreases in matrix metalloproteinase (MMP), cholinesterase (ChE), and adenosine triphosphate (ATP) levels; alleviates liver oxidative stress; reduces blood glucose and lipid levels; decreases alanine transaminase (ALT) and aspartate aminotransferase (AST) activity; reduces the liver index; and significantly decreases the degree of hepatic steatosis (Yong-Jin et al., 2011). Geniposide increases metabolism, enhances insulin sensitivity and alleviates oxidative stress through many mechanisms. Oxidative stress can activate multiple stress-sensitive pathways, including the nuclear transcription factor-κB (NF-κB), p38 mitogen-activated protein kinase (p38MAPK) and c-Jun amino-terminal kinase/stress-activated protein kinase (JNK/SAPK) pathways, impairing insulin signaling processes (Newsholme et al., 2007). Geniposide can restore the function of these pathways by reversing JNK overactivation and decreasing protein kinase B (AKT/PKB) phosphorylation, thereby alleviating IR by inhibiting oxidative stress and ameliorating mitochondrial dysfunction, effects that are particularly beneficial in the elderly population (Zhuge et al., 2023). Additionally, geniposide increases glucose-stimulated insulin secretion by increasing the level of glucose transporter protein 2 (GLUT2), which activates glucagon-like peptide-1 (GLP-1) in pancreatic β-cells via a phosphatidylinositol 3-kinase (PI3K)-dependent mechanism (Guo et al., 2014). In summary, geniposide ameliorates IR through multiple mechanisms, and its antioxidant properties can reduce the damage caused by oxidative stress via the insulin signaling pathway, regulate blood glucose and blood lipid levels, and promote the function of pancreatic islet cells in various ways.

Geniposide also modulates insulin signaling-related molecules, enhances glucose uptake by increasing insulin receptor substrate-1 (IRS-1) and GLUT1 protein levels, and blocks the IR-induced phosphorylation of IRS-1 and AKT Thr308. Thioredoxin-interacting protein (Txnip) is closely related to IR. Geniposide promotes Txnip degradation through the proteasomal pathway while inducing adenosine 5‘-monophosphate (AMP)-activated protein kinase (AMPK) phosphorylation under high-glucose conditions (Zhao et al., 2021). Therefore, geniposide may alleviate IR in hepatic adipocytes through AMPK-mediated Txnip degradation (Zhao et al., 2021). Hormone-sensitive lipase (HSL) is a key rate-limiting enzyme that regulates triglyceride (TG) mobilization. Adipose triglyceride lipase (ATGL) specifically hydrolyses the first ester bond in TGs. Geniposide promotes lipolysis by increasing HSL and ATGL expression in a dose-dependent manner. In addition to lipolysis, FFA accumulation in the circulation and in tissues and organs leads to lipid accumulation and IR in peripheral tissues; however, fat accumulation and IR symptoms are significantly reduced after geniposide treatment (Guan et al., 2018). Wnt signaling is an important regulator of adipose tissue development, and Wnt/β-catenin signaling is a key factor in β-cell insulin secretion (Rulifson et al., 2007) and growth and regeneration (Figeac et al., 2010). The overexpression of transcription factor 7 like 2 (TCF7L2), a major transcription factor of Wnt signaling, promotes the expression of GLP-1R (Shu et al., 2009). Geniposide activates the Wnt/β-catenin signaling pathway by upregulating the expression of TCF7L2 mRNA and activating GLP-1R to stimulate insulin secretion. Therefore, geniposide promotes the regeneration and survival of β-cells, stimulates insulin secretion, lowers blood glucose levels, and ameliorates hepatic cell steatosis by activating the β-catenin/TCF7L2 signaling pathway (Yao et al., 2015). In summary, geniposide attenuates IR through multiple mechanisms, including the regulation of insulin signaling-related molecules, the enhancement of glucose uptake, the regulation of lipid metabolism through lipolytic enzymes, and the promotion of β-cell regeneration in multiple ways.

## 3 Geniposide prevents oxidative stress

Under physiological conditions, a dynamic balance is maintained between oxidation and antioxidation in the human body. Oxidative stress results in excessive ROS production, which leads to the generation of polyunsaturated fatty acids that promote proteolysis, decreased secretion of low-density lipoprotein (LDL), and the accumulation of TGs in the liver (Leung et al., 2011). According to the two-hit theory, IR is the first hit, and oxidative stress is the second hit. Under IR, fatty acid β-oxidation is accelerated, and a large amount of ROS are generated. When the ROS content exceeds the cellular antioxidant capacity, oxidative stress is triggered, leading to the accumulation of peroxides, which further exacerbates steatosis.

Nuclear factor erythroid 2-related factor 2 (Nrf2) is a transcription factor that regulates cellular redox reactions. During oxidative stress, Nrf2 binds to antioxidant response elements (AREs) and regulates the downstream expression of the antioxidant protein heme oxygenase-1 (HO-1), which catalyzes the degradation of hemoglobin, forming an endogenous antioxidant protection system, whereas PI3K controls the transport activity (Kang et al., 2002) and glycogen synthase kinase 3β (GSK3β)-mediated degradation of Nrf2 (Salazar et al., 2006). Therefore, NAFLD patients often exhibit decreased Nrf2 levels. Geniposide is a potent antioxidant that scavenges reactive oxygen species and free radicals and has antioxidant and protective effects on mitochondrial function (Buzzetti et al., 2016). It may eliminate ROS damage by activating the Nrf2 signaling pathway and promoting the expression of antioxidant enzymes such as glutathione (GSH) and HO-1 (Okada et al., 2007). HO-1 is a potent endogenous antioxidant gene, and the induction of HO-1 can reduce lipid deposition and prevent the development of liver fibrosis (Sodhi et al., 2015). A previous study revealed that geniposide can significantly reduce ROS levels, and increase the activity of the antioxidant glutathione peroxidase (GSH-Px) while elevating the expression of Nrf2 and HO-1 under hypoxic and hyperglycemic conditions, maintain a state of redox equilibrium and reduce oxidative stress-related damage through the modulation of the Nrf2/HO-1 signaling pathway, which provides a theoretical basis for the use of geniposide as an antioxidant to treat NAFLD (Dong et al., 2022). Moreover, geniposide reduces fatty acid and sterol synthesis, ameliorates oxidative stress and alleviates NAFLD by upregulating Nrf2 expression and regulating the protein expression of components of the AMPK/PI3K/mammalian target of rapamycin (mTOR) signaling pathway, inhibiting the phosphorylation of mTORC and its related proteins and modulating the expression of sterol regulatory element binding protein-1c (SREBP-1c) (Shen et al., 2020). Geniposide can directly inhibit SREBP-1c expression and fatty acid synthase expression to inhibit lipid synthesis (Zhong et al., 2018a). The induction of inducible nitric oxide oxidation synthase (iNOS) further enhances oxidative stress in damaged tissues (Liu et al., 2018). Geniposide inhibits NO release and reduces iNOS protein and mRNA expression levels by suppressing two signaling pathways that induce iNOS, namely, the nuclear factor-κB inhibitory protein (I-κB) degradation/NF-κB pathway and the interleukin-1 (IL-1) type I receptor pathway (Nakatake et al., 2017).

The hyperactivation of hypoxia-inducible factor (HIF) promotes NADPH oxidase-2 (NOX2) expression, increases ROS levels, and inhibits superoxide dismutase (SOD), leading to oxidative stress (Huang et al., 2016). HIF can also directly regulate the expression of 1-acylglycerol-3-phosphate O-acyltransferase 2 (AGPAT2), which is involved in triacylglycerol biosynthesis and induces lipid synthesis (Triantafyllou et al., 2018). The proliferation of inflammatory cells and oxidative stress induced by advanced glycosylation end products (AGEs) promote steatosis and accelerate NAFLD. An increase in AGE levels induces the production of receptor for advanced glycosylation end products (RAGE) and activates AGE-RAGE signaling (Litwinoff et al., 2015). Geniposide may alleviate oxidative stress, inhibit fat synthesis, and slow the progression of simple steatosis to NAFLD by modulating the HIF-1 and AGE-RAGE signaling pathways (Sun et al., 2024).

The liver is a major organ for fatty acid β-oxidation. PPARα is a major regulator of hepatic β-oxidation and microsomal ω-oxidation, and induces a decrease in the expression of hepatic steatosis-associated substances by enhancing mitochondrial β-oxidation. Carnitine palmitoyltransferase 1α (CPT-1α) is a key regulator of mitochondrial β-oxidation. Decreased PPARα expression leads to hepatic lipid accumulation, whereas PPARα activation increases the expression of fatty acid oxidation-related genes and reduces the risk of hepatic steatosis. Geniposide promotes lipolysis and fatty acid β-oxidation, promotes lipoprotein lipase (LPL) and apolipoprotein A (Apo A) expression, and reduces fatty acid synthetase expression by upregulating PPARα and CPT-1α gene expression in hepatic tissues, which reduces cholesterol and TG production and ameliorates hepatic steatosis (Guan et al., 2018). The overexpression of cytochrome P450 2E1 (CYP2E1) results in the generation of a large amount of ROS, leading to lipid peroxidation, impairing biofilm function and inducing NAFLD. Geniposide inhibits the expression of CYP2E1 and increases the expression of PPARα (Ma et al., 2011). In summary, gardenia glycosides have multiple positive effects through their antioxidant activity. On the one hand, they promote lipolysis and fatty acid β-oxidation in liver tissue by increasing the expression of PPARα and CPT-1α and suppress oxidation that may be triggered by lipid accumulation; on the other hand, gardenia jasmine glucosides can simultaneously inhibit the expression of CYP2E1, reduce the generation of ROS, and increase the expression of PPARα, in addition to elevating the antioxidant capacity of the liver via multiple synergistic effects.

## 4 Geniposide alleviates mitochondrial dysfunction

During the progression of NAFLD, hepatic mitochondrial ribonucleic acid (mtRNA) is sensitive to oxidative stress and lacks histone protection, and excessive ROS attack respiratory chain complexes and mtDNA and damage the mitochondrial ultrastructure, leading to mitochondrial dysfunction, which in turn affects hepatic metabolism, forming a vicious cycle (Lin et al., 2013). Moreover, toxic lipids may impair mitochondrial function, and in the presence of increased fat deposition in liver tissue, excessive oxidative stress may cause mitochondrial swelling or destruction, leading to mitochondrial dysfunction and ROS spillover. Mitochondrial structural defects and impaired function have been observed in patients with nonalcoholic steatohepatitis (NASH) (Sanyal et al., 2001). UCP2, an inner mitochondrial membrane protein with proton channel function, leaks H^+^ through the mitochondrial membrane into the matrix, reduces ATP synthesis, and decreases ROS production by lowering the mitochondrial proton gradient and membrane potential to prevent oxidative stress (Nesci and Rubattu, 2024). However, oxidative stress can promote UCP2 expression, which causes mitochondrial damage, promotes the necrosis of damaged hepatocytes and exacerbates NAFLD progression (Nesci and Rubattu, 2024; Fan et al., 2020).

Geniposide is an *in vivo* inhibitor that specifically targets UCP2; it freely crosses the cell membrane and inhibits UCP2-mediated proton leakage in mitochondria, increases the mitochondrial membrane potential, increases ATP levels, and closes K (ATP) channels without affecting other mitochondrial functions (Zhang et al., 2006). Geniposide increases the mtDNA copy number and alleviates the impairment of mitochondrial dynamics and autophagy. In addition, the combination of geniposide with VitE significantly reduces UCP2, NF-κB and tumor necrosis factor-α (TNF-α) protein expression; increases the mitochondrial membrane potential; and suppresses hepatic lipid production (Lin et al., 2010). However, a previous study revealed that geniposide induces apoptosis by inhibiting signal transducer and activator of transcription factor 3/myeloid cell leukemia sequence 1 (Stat3/Mcl-1) pathway, leading to mitochondrial dysfunction (Zhuge et al., 2023). The upregulation of UCP2 expression has both beneficial and detrimental effects. On the one hand, it reduces intracellular ATP production through proton leakage, thus increasing the sensitivity of hepatocytes to damaging factors and promoting hepatocyte damage and death; on the other hand, it reduces lipogenesis through a reduction in intracellular ROS production, thus playing a protective role in the liver (Ma et al., 2012).

UCP2 has been demonstrated to play dual roles in the development of NAFLD. On the one hand, the upregulation of UCP2 has been shown to protect the liver from lipotoxic damage by reducing ROS production in mitochondria and preventing lipid peroxidation. For example, in mice with high-fat diet (HFD)-induced NAFLD, a reduction in the level of UCP2 induced by small interfering RNA (siRNA) resulted in significant cellular pyroptosis (Zhong et al., 2018b), suggesting a protective role for reduced UCP2 expression in the early stages of the disease. Conversely, sustained activation of UCP2 has been demonstrated to result in impaired energy metabolism, as excessive proton leakage has been shown to reduce ATP production and promote apoptosis in hepatocytes under prolonged metabolic stress. Geniposide, a natural inhibitor of UCP2, has been shown to ameliorate hepatic steatosis by blocking UCP2-mediated proton leakage and increasing the mitochondrial membrane potential and ATP synthesis. However, it has been demonstrated that the inhibition of UCP2 by geniposide may result in an increase in mitochondrial ROS levels when cells are already under oxidative stress (Habtemariam and Lentini, 2018). These findings indicate that the efficacy of geniposide is contingent upon the redox state of the hepatocyte microenvironment. Subsequent studies should focus on the temporal dynamics of the inhibitory effect of geniposide on UCP2, determining whether geniposide exerts this inhibitory effect during the early stage of NAFLD, when ROS levels are reduced, or during the late stage of NAFLD, when a substantial amount of ATP is consumed. Therefore, whether geniposide can be used as a treatment for NAFLD needs to be thoroughly investigated in clinical trials.

## 5 Geniposide suppresses the inflammatory response

Infection and cell death are the two central factors involved in the development of inflammation. Pathogen-associated molecular patterns (PAMPs) (Gao et al., 2019) and damage-associated molecular patterns (DAMPs) (Yang and Tonnesseen, 2019) trigger the inflammatory response through toll-like receptors (TLRs) and inflammatory factors, respectively, leading to the transcription of proinflammatory factors. NF-κB transcription factor family members are considered central mediators of inflammatory processes that regulate the expression of proinflammatory cytokines (DiDonato et al., 2012); TNF-α and IL-1β, the main proinflammatory factors in the toll-like receptor 4 (TLR4)/NF-κB signaling pathway, can activate immune cells, aggravate the inflammatory response and liver injury (Jaeschke, 2015), promote the synthesis and release of chemokines, and recruit immune cells to the damaged area, further amplifying the inflammatory response and aggravating liver injury.

Kupffer cells, as hepatic macrophages, amplify inflammatory effects by continuously releasing proinflammatory factors and chemokines. Kupffer cell activation is dependent on the TLR4/NF-κB signaling pathway, and geniposide attenuates intrahepatic neutrophil and macrophage infiltration and reduces inflammatory responses by inhibiting the TLR4/NF-κB signaling pathway (Yang et al., 2019). TLR4 is a key sensor for transmitting inflammatory signals and stimulating the release of inflammatory mediators. NF-κB and mitogen-activated protein kinases (MAPKs) activate proinflammatory cytokine production (Lu et al., 2008). MAPK activation in response to stress impairs insulin regulation and lipid metabolism. TLR4 activates downstream medullary differentiation factor 88 (MyD88) through the initiation of the NF-κB and MAPK signaling pathways (Muzio et al., 1997), mediating proinflammatory cytokine release. Geniposide significantly inhibits the expression of TLR4, i-κB-α, NF-κB, p38, and extracellular signal-regulated kinase (ERK) and the phosphorylation of JNK, thereby inhibiting the downstream NF-κB and MAPK signaling pathways and the release of the inflammatory factors TNF-α, IL-1β, and IL-6 (66). iNOS and COX-2 are also involved in the inflammatory process, regulating the expression of the inflammatory mediators NO and prostaglandin E2 (PGE2) (Zwolinska-Wcislo et al., 2011). Some experiments have shown that geniposide can significantly reduce the levels of NF-κB, iNOS, and COX-2 and decrease the levels of proinflammatory cytokines (TNF-α, IL-1β, and IL-6) and myeloperoxidase (MPO) activity in rats, thus effectively inhibiting the infiltration of inflammatory factors (Xu et al., 2017). In conclusion, geniposide inhibits key inflammatory signaling pathways, such as the TLR4/NF-κB and MAPK pathways; suppresses the release of inflammatory factors, such as TNF-α, IL-1β and IL-6; and decreases the levels of iNOS and COX-2; thus, geniposide can effectively inhibit the infiltration of inflammatory cells in the liver and represents a therapeutic agent for NAFLD.

The myosin light chain kinase (MLCK) protein participates in the inflammatory response by regulating cytoskeletal contraction and endothelial cell permeability, whereas the secretion of inflammatory factors affects the expression of tight junction proteins, which further contributes to the progression of inflammation. Geniposide inhibits aberrant MLCK and tight junction protein expression and activates 5'-AMP-activated protein kinase phosphorylation (Shan et al., 2017). AMPK, a kinase that regulates energy homeostasis, can simultaneously regulate fatty acid synthesis, autophagy, and signaling pathways (Herzig and Shaw, 2018). Research has shown that geniposide can ameliorate inflammatory responses and modulate barrier dysfunction by activating the AMPK pathway (Xu et al., 2017). The activation of NOD-like receptor pyrin domain-containing protein 3 (NLRP3) and NLR family CARD-containing protein 4 (NLRC4) can lead to hepatocyte pyroptosis and the release of inflammatory factors, which promote hepatic inflammation and fibrosis and induce the development of NAFLD. Studies have shown that geniposide exerts anti-inflammatory effects by inhibiting the activation of the NLRP3 and NLRC4 inflammasomes and inhibits the formation of the apoptosis-associated speck-like protein (ASC) complex induced by NLRP3 agonists without affecting the NLRC4 agonist-induced formation of the ASC complex, suggesting that geniposide may affect upstream processes such as autophagy (Yu et al., 2015). Geniposide has been shown to inhibit NLRP3 inflammatory vesicle activation both *in vivo* and *ex vivo* (Yu et al., 2015) and reduce ATP- and hydrogen peroxide (H_2_O_2_)-mediated secretion of IL-1β (Rajanbabu et al., 2015), suggesting its potential as a therapeutic agent for NAFLD.

## 6 Geniposide resists apoptosis

Hepatocyte death is a key factor in the progression of liver disease, causing hepatitis, liver fibrosis, cirrhosis, and hepatocellular carcinoma (Luedde et al., 2014). Apoptosis mediated by the Fas/Fas ligand (FasL) pathway is one of the mechanisms involved in the pathogenesis of NAFLD, and the ROS-induced binding of FasL to the Fas receptor (FasR) in hepatocytes triggers apoptosis, which activates Kupffer cells and hepatic stellate cells (HSCs) and promotes hepatic fibrosis (Lin et al., 2013). In hepatocytes, some lipids, such as FFAs, can lead to lipotoxicity and thus apoptosis. Apoptotic vesicles generated after hepatocyte apoptosis are phagocytosed by hematopoietic stem cells and stimulate cellular fibrosis, whereas DNA fragments from apoptotic cells can also activate hematopoietic stem cells to promote hepatic fibrogenesis (Watanabe et al., 2007).

It has been previously reported that UCP2 promotes the necrosis of damaged hepatocytes. Geniposide reverses HFD-induced liver injury and inhibits UCP2-mediated hepatocyte death (Zhong et al., 2018b). Geniposide can also slow the progression of NAFLD by negatively regulating apoptosis. Many studies have suggested that geniposide may act through different targets; however, this remains unproven experimentally and clinically. mTOR is an autophagy-related gene, and changes in the phosphorylation level of the mTOR protein can regulate the downstream activation of autophagy signaling pathways, and increased levels of mTOR phosphorylation can inhibit cellular autophagy (Al-Bari and Xu, 2020). AMPK is upstream of mTOR, and AMPK can inhibit mTOR, which indirectly activates cellular autophagy (Arsikin et al., 2012). Geniposide inhibits AMPK phosphorylation and activates mTOR phosphorylation by blocking the AMPK/mTOR signaling pathway in the regulation of autophagy in hepatocytes, thereby inhibiting cellular autophagy (Liu et al., 2021). Apoptosis is regulated by Bax, Bcl-2 and caspase family members (Tian et al., 2015). Bax and Bcl-2 are antagonistic homologous factors that regulate apoptosis. The activation of Bax results in pore formation, leading to a loss of membrane integrity (Tait and Green, 2010), whereas Bcl-2 inhibits the function of Bax and decreases the Bax/Bcl-2 ratio, which affects the level of apoptosis. Increased Bax expression activates caspase-9, which in turn activates caspase-3, leading to apoptosis (Perumal et al., 2018; Zhu et al., 2012). Geniposide decreases Bax, caspase 3, and caspase 9 expression and increases Bcl-2 expression (Yang et al., 2021). P53 activates Bax expression, blocks Bcl-2 expression, and activates caspase-3 to induce apoptosis. In addition, TLR4 plays a dominant role in LPS-induced apoptosis, and its activation triggers downstream apoptotic signaling pathways. Geniposide blocks apoptotic signaling by downregulating TLR4 expression and inhibiting p53 phosphorylation to downregulate Bax expression and upregulate Bcl-2 expression (Song et al., 2014b). Moreover, geniposide inhibits hepatocyte apoptosis by regulating the overall DNA methylation level; modulating the expression levels of p53, Bcl-2, and AKT; and decreasing the DNA methylation of the AKT and Bcl-2 promoter regions (Peng et al., 2023). Here, we summarize recent studies on the treatment of fatty liver with geniposide in Table 1.

**TABLE 1:** Summary of key findings of studies on the main mechanisms by which geniposide alleviates NAFLD.

Effect	Cells/animals	Modeling method	Mechanisms	Ref.
Amelioration of insulin resistance	Male Sprague–Dawley rats (3 and 18 months old)	/	Stimulates insulin secretion from pancreatic islet cells to ameliorate insulin sensitivity, inhibits excessive ROS production, attenuates the reduction in the MMP and ATP levels, reverses oxidative stress-associated JNK hyperactivation and reduces AKT phosphorylation	Guan et al. (2013)
	3T3-L1 mouse preadipocytes	Treatment with a standard differentiated medium containing 1.0 μg/mL insulin, 0.25 μM DEX, 0.5 mM IBMX, 2.5 μM rosiglitazone, ABP (1:1000), and 1 nM T3 for 7 days and then incubation with 1.0 μg/mL insulin, 2.5 μM rosiglitazone, ABP (1:1000), and 1 nM T3 for 4 days	Increases IRS-1 and GLUT1 expression levels, enhances glucose uptake, and prevents IR-induced phosphorylation of IRS-1 and AKT (Thr308); promotes Txnip degradation via the AMPK pathway	Zhao et al. (2021)
	INS-1 cells	Treatment with 10 µM LY294002	Increases the levels of GLUT2, and activates GLP-1 in pancreatic β-cells through a PI3K-dependent mechanism	Guo et al. (2014)
	Male Sprague–Dawley rats (6 weeks old)	Feeding of a high‐fat diet containing 30% (w/w) lard	Upregulates the gene expression of HSL and ATGL to promote lipolysis	Guan et al. (2018)
	Four-week-old male C57BL/6J mice	Feeding of a high-fat diet or normal chow diet	Upregulates the mRNA expression of TCF7L2 to activate the Wnt/β-catenin signaling pathway, which activates GLP-1R expression and stimulates insulin secretion	Yao et al. (2015)
Antioxidative stress	Male Sprague‒Dawley rats	Administration of ICKT	Activates the Nrf2/HO-1 signaling pathway and promotes the expression of several antioxidant enzymes including GSH and HO-1	Okada et al. (2007)
	Male wild‐type (WT) and Nrf2^−/−^ C57BL/6 mice (6–8 weeks old)	Treatment with tyloxapol	Upregulates the protein expression of Nrf2 and components of the AMPK/PI3K/mTOR signaling pathways, thereby inhibiting the phosphorylation of mTORC and associated proteins and regulating SREBP-1c expression to reduce fatty acid and sterol synthesis	Shen et al. (2020)
	Fifty male C57BL/6J mice (6–8 weeks old)	Feeding of a high-fat diet	Directly inhibits SREBP-1c expression and suppresses lipid synthesis	Zhong et al. (2018a)
	Rat hepatocytes	Stimulation with IL-1β	Inhibits NO release; suppresses the expression of components of two signaling pathways, I-κB degradation, NF-κB activation and the upregulation of type I IL-1 receptor; and reduces iNOS protein and mRNA expression levels	Nakatake et al. (2017)
	Four-week-old C57/BL6 male or female mice	Induction of hyperglycemia by injecting streptozotocin (STZ) solution	Regulates the HIF-1 and AGE-RAGE signaling pathways	Sun et al. (2024)
	Male Sprague–Dawley rats (6 weeks old)	Feeding of a high‐fat diet	Upregulates the gene expression of PPARα and CPT-1α, promotes lipolysis and fatty acid β-oxidation, promotes the expression of LPL and Apo A, reduces the expression of fatty acid synthesis-related enzymes, and reduces cholesterol and triglyceride production	Guan et al. (2018)
	Male Sprague–Dawley rats	Induction of NASH by oral administration of a high-fat emulsion	Inhibits CYP2E1 overexpression and reduces ROS generation	Ma et al. (2011)
Improvement of mitochondrial dysfunction	Male ob/ob mice (16–20 weeks old)	UCP2 knockout	Inhibits UCP2-mediated proton leakage, increases the mitochondrial membrane potential, elevates ATP levels and closes K (ATP) channels	Zhang et al. (2006)
	L02 cells	/	In combination with VitE, significantly reduces UCP2, NF-κB and TNF-α protein expression and increases the mitochondrial membrane potential	Lin et al. (2010)
Anti-inflammatory response	Male C57 mice weighing 18–22 g (6–8 weeks old)	Induction of acute liver injury by intraperitoneal injection of APAP	Inhibits the TLR4/NF-κB signaling pathway, attenuates damage caused by the infiltration of intrahepatic neutrophils and macrophages, and reduces the inflammatory response	Yang et al. (2019)
	BALB/c mice that (6–8 weeks old, 50 female and 25 male)	Induction of mastitis by LPS	Inhibits TLR4 expression and i-κB-α, NF-κB, p38, ERK, and JNK phosphorylation, which in turn inhibits the downstream NF-κB and MAPK signaling pathways and the release of the inflammatory factors TNF-α, IL-1β, and IL-6	Song et al. (2014a)
	Male Sprague‒Dawley rats	Induction of colitis by rectal administration of TNBS	Downregulates the expression of the NF-κB, COX-2, iNOS and MLCK proteins, reduces the levels of TNF-α, IL-1β and IL-6 and the MPO activity, and inhibits the infiltration of inflammatory factors; activates the AMPK signaling pathway, reduces inflammation and regulates the function of the damaged epithelial barrier	Xu et al. (2017)
	NLRP3^−/−^ and C57BL/6J mice	Induction of BMDM by LPS and alum	Inhibits NLRP3 and NLRC4 inflammasome activity	Yu et al. (2015)
Anti-apoptosis	Eight-week-old male ApoE^−/−^ mice	Induction of AS by feeding of a HFD for 12 weeks	Blocks the AMPK/mTOR signaling pathway to regulate cellular autophagy, inhibits AMPK phosphorylation and activates mTOR phosphorylation to inhibit cellular autophagy	Liu et al. (2021)
	Male C57BL/6 mice	Induction of liver fibrosis via intraperitoneal injection of CCl_4_	Decreases the expression of Bax, caspase 3, caspase 9 and elevates the expression of Bcl-2	Yang et al. (2021)
	BALB/c mice (6–8 weeks old, 80 female and 40 male)	Induction of infection of the mammary gland	Downregulates TLR4 expression, inhibits p53 phosphorylation to downregulate Bax expression and upregulates Bcl-2 expression to block apoptotic signaling	Song et al. (2014b)
	L-02 normal human hepatocytes	Culture in RPMI-1640 medium supplemented with 10% fetal bovine serum, 100 U/mL penicillin and 100 μg/mL streptomycin	Reduces the DNA methylation of the AKT and Bcl-2 promoter regions by regulating overall DNA methylation levels and modulating the expression levels of p53, Bcl-2 and AKT	Peng et al. (2023)

## 7 Geniposide toxicity

Although geniposide has a wide range of biological activities and acts against various mechanisms of NAFLD, its toxicity needs to be addressed. Hepatotoxicity is a fundamental issue that affects the safety of geniposide. A study in rats on the potential hepatotoxicity of geniposide revealed that high doses of geniposide (574 mg/kg or more) causes acute hepatotoxicity after 24–48 h of oral administration, an effect that may be related to oxidative stress, whereas normal doses of geniposide (24.3 mg/kg or less) do not cause hepatotoxicity, even when repeatedly administered for 90 consecutive days (Ding et al., 2013). In addition to normal rats, rats with α-naphthyl isothiocyanate (ANIT)-induced cholestasis also exhibit hepatotoxicity after geniposide administration, resulting in severe pathological damage to the liver. A recent study revealed potential adverse effects, including hepatic injury, associated with geniposide and its metabolite genipin, particularly high doses of genipin or the long-term treatment with genipin. Therefore, for optimal therapeutic effects, the duration of treatment needs to be reduced when the dose is increased (Qiu et al., 2024). Owing to the hepatotoxicity of geniposide, especially at high doses or under specific pathological conditions, it is necessary to manage the dose and duration of treatment to ensure safety and efficacy when it is applied to treat NAFLD.

In addition, high-dose and prolonged administration of geniposide has also been associated with nephrotoxicity (Liu et al., 2022). Furthermore, gastrointestinal adverse effects are another critical concern during geniposide application. Absorption of geniposide into the mesenteric veins may contribute to idiopathic mesenteric phlebosclerosis (IMP) (Young and Yeh Lee, 2023), whereas oral administration of the yellow pigment of gardenia has been reported to induce diarrhea and pathological alterations in the intestinal tract, particularly in the duodenum (Yamano et al., 1988). When evaluating the side effects of geniposide, the influence of the dosage must be thoroughly considered. Geniposide has been demonstrated to exhibit both insulin-stimulating and anti-inflammatory properties. However, high doses of geniposide may induce hypoglycemia, particularly in patients with both diabetes and NAFLD. Moreover, prolonged immunosuppressive effects could compromise host immune responses. Consequently, the potential toxicity of geniposide should be thoroughly evaluated to assess its feasibility for clinical application.

## 8 Conclusions and future perspective

**FIGURE 2:**
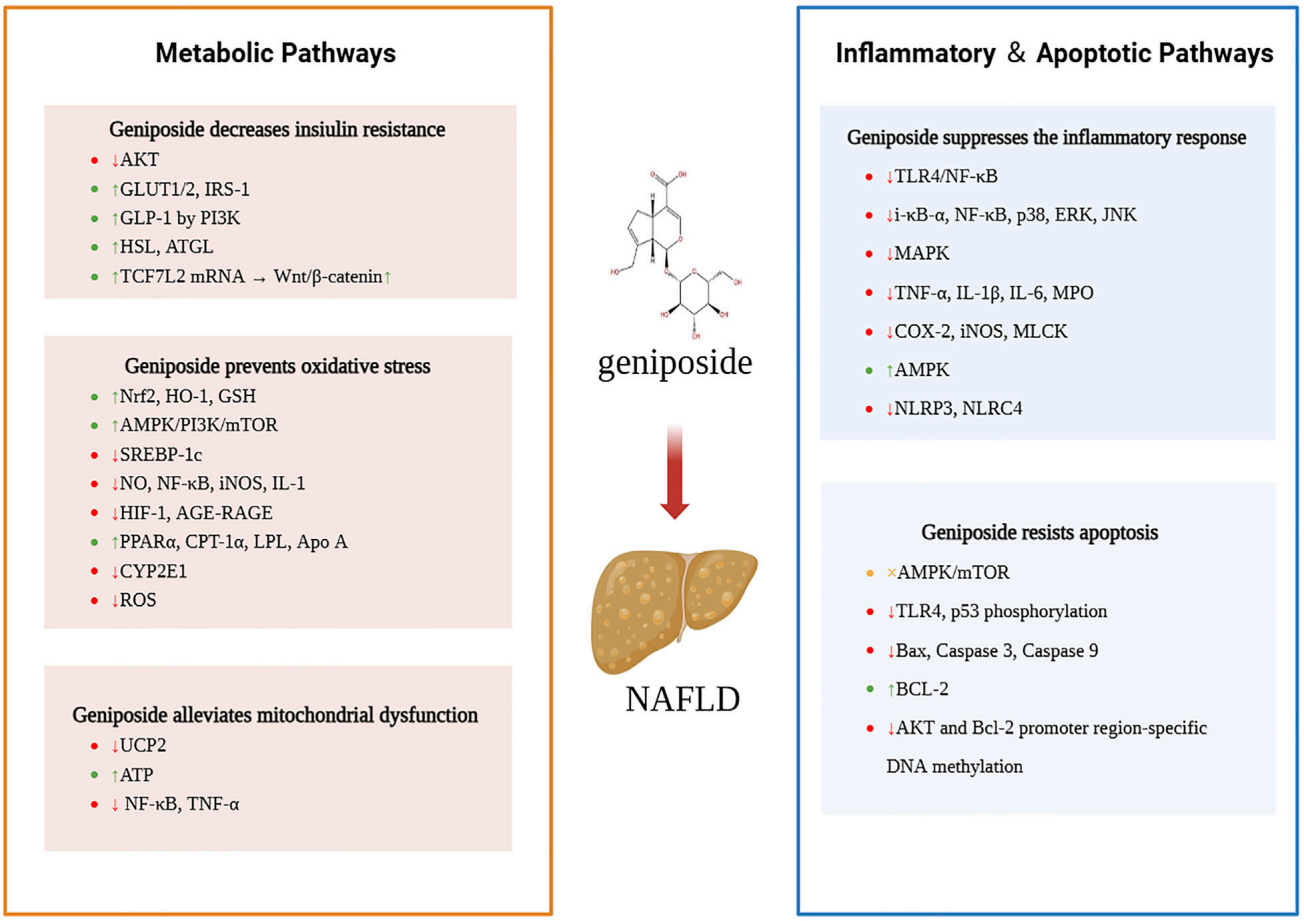
Mechanisms by which geniposide ameliorates NAFLD.

In recent years, the pharmacological effects of geniposide have attracted the attention of researchers. Numerous studies have confirmed that geniposide, the main pharmacological component of *Gardenia jasminoides*, can target different mechanisms of NAFLD, indicating its great potential for the treatment of liver diseases (Figure 2). In contrast to other drug candidates for NAFLD, such as FXR activators (e.g., obeticholic acid, OCA), which act through a single target to regulate bile acid metabolism (Adorini and Trauner, 2023), geniposide has been shown to have broad-spectrum therapeutic potential, as it exerts synergistic therapeutic effects, such as anti-inflammatory and antioxidant effects, through multiple pathways. Despite the challenges posed by its pleiotropic nature, which complicates the elucidation of its mechanism of action and leads to a propensity for off-target effects (the inhibition of UCP2 by elevated doses of geniposide exacerbates oxidative stress), the utilization of gardenia extract as a natural constituent may mitigate the risk of immunogenicity and circumvent the adverse effects of OCA, such as itchiness and elevated LDL levels (Wang et al., 2024).

This review systematically integrates for the first time the pathways through which geniposide targets multiple pathogenetic mechanisms of NAFLD and comprehensively reveals the intrinsic connection of geniposide with the regulation of signaling factors at the cellular level (e.g., inhibition or activation of specific signaling pathways such as the TLR4/NF-κB pathways) to improve function at the tissue and organ levels (e.g., alleviation of hepatic fibrosis). This overcomes the limitations of previous studies that were limited to a single pathogenic mechanism, provides a framework for exploring the innovative application of geniposide in NAFLD treatment, and promotes its rapid translation from basic research to clinical practice.

Nevertheless, this review has several limitations. First, the majority of the studies referenced in the article were based on experimental animal and cell models, so the validity and safety of extrapolating the conclusions to clinical settings are limited. Differences in metabolic regulation between humans and mice, including variations in bile acid synthesis and diverse enzyme activities, may lead to variations in the pharmacokinetics and pharmacodynamics of geniposide in the two species. Additionally, the majority of animal models (e.g., the high-fat diet-induced NAFLD mouse model) replicate a single aspect of NAFLD pathogenesis but do not fully recapitulate the complex pathogenesis observed in humans. Consequently, conclusions related to the effectiveness of geniposide in the treatment of NAFLD must be interpreted with caution and repeatedly validated in NAFLD patient populations.

Second, the oral bioavailability of geniposide is suboptimal. Geniposide is rapidly absorbed into the systemic circulation and eliminated from the plasma within 12 h. As it does not readily permeate the intestinal membrane, it is easily hydrolyzed to genipin in the intestine, and sulfation occurs in the intestine and liver. Consequently, its absolute oral bioavailability is low, at only 9.67%, and its bioavailability is low (Wang et al., 2014). Moreover, hydrolysis and first-pass metabolism by the intestinal microbiota also reduce its systemic levels (Jin et al., 2014). To address this issue and increase its bioavailability, solid lipid nanoparticles have been used as to deliver geniposide to improve its bioavailability (Wang et al., 2014). Furthermore, the incorporation of geniposide into liposomes can significantly extend its half-life and enhance its ability to target the brain (Wan et al., 2021). In addition, herb–herb combinations, such as the combination of Gardenia jasminoides Ellis (ZZ) with Fructus aurantii immaturus (ZS) and/or Cortex magnoliae officinalis (HP), may increase the absorption of geniposide and increase its oral bioavailability (Sun et al., 2012). When geniposide is included in compound formulas, such as Huang-Lian-Hou-Pu decoction (HLHPD), Huang-Lian-Jie-Du decoction (HLJDD) and Huang-Lian-Zhi-Zi decoction (HLZZD), its half-life is shortened, and the AUC and oral bioavailability are increased (Liu et al., 2022). Although the mechanism underlying the multitarget effects of geniposide provides a sufficient theoretical basis for its use to treat NAFLD, its potential for clinical application still depends on increasing its bioavailability, the success of clinical human trials, and the identification of the ideal dosage range. In summary, further in-depth is needed to increase the applicability of geniposide, a natural drug.
